# An automated GC/MS system for the analysis of volatile and semi-volatile organic compounds in water

**DOI:** 10.1155/S1463924693000173

**Published:** 1993

**Authors:** Allen K. Vickers, Lowell M. Wright

**Affiliations:** OI Analytical PO Box 9010 College Station Texas 77842-9010 USA

## Abstract

This paper describes a GC/MS system capable of performing Volatile Organic Analysis on liquids, solids, and air. When combined with a syringe auto-injector, the system is completely automated for both volatile and semi-volatile analyses. An OI Analytical Model 4551 Vial Multisampler and an OI Analytical DPM-16 Multisampler are interfaced and then connected to an OI Analytical Model 4560 Sample Concentrator, an HP Model 5971 MSD, an HP Model 7673 Auto-Injector, and an HP Model 5890 Series II GC to form a multi-tasking GC/MS system. This system is shown to allow greater versatility in the laboratory.


Journal of Automatic Chemistry, Vol. 15, No. 4 (July–August 1993), pp. 133–139

### An automated GC/MS system for the analysis of volatile and semi-volatile organic compounds in water

**Allen K. Vickers and Lowell M. Wright**
*OI Analytical, PO Box 9010, College Station, Texas 77842-9010, USA*

*This paper describes a GC/MS system capable of performing Volatile Organic Analysis on liquids, solids, and air. When combined with a syringe auto-injector, the system is completely automated for both volatile and semi-volatile analyses. An OI Analytical Model 4551 Vial Multisampler and an OI Analytical DPM-16 Multisampler are interfaced and then connected to an OI Analytical Model 4560 Sample Concentrator, an HP Model 5971 MSD, an HP Model 7673 Auto-Injector, and an HP Model 5890 Series II GC to form a multi-tasking GC/MS system. This system is shown to allow greater versatility in the laboratory.*

### Introduction

For today's laboratory needs, multi-tasking equipment is increasingly necessary, particularly for gas chromatography/mass spectrometry (GC/MS) systems. Equipping a GC/MS system to perform a single analysis is costly but necessary when protocol requires a mass spectrometer detector (MSD) for positive analyte identification. Not only is a suitable GC/MS system needed (these have been available for some time), but also the ability to create a multi-tasking system. It is necessary to interface the required apparatus to perform a variety of analyses.

Until recently, USEPA Method 524.2 or 624 requirements have limited the GC/MS system by requiring that the GC injector interface to a purge-and-trap sample concentrator. This has become less of a limitation with the appearance of certain interfaces (such as the Low-Dead-Volume Injector) on the market, which allow more utility of the system. However, the weaknesses of these systems are related more to sample introduction than to GC. There is a need to analyse a variety of matrices (for example water, soil, extractables, air) by sample concentration. Using the GC/MS system for more than volatiles-only analyses is also desirable (for example, for base neutral acids (BNA) analysis). This requires using a syringe autosampler, combined with a sample concentrator, creating several multisampling capabilities.

The GC/MS system described in this paper demonstrates such capabilities. It incorporates the use of a capillary column (interfaced directly to the MS), which separates a wide range of analytes and has a large temperature range. The inlet is a standard split-splitless (S/SL) injection port. More importantly, the laboratory can perform a wide range of volatile and semi-volatile organic analyses using this new multi-tasking system.

### Description of system

Figure 1 shows the complete system used. The GC was a Model 5890 Series II (Hewlett-Packard, Little Falls, DE, USA). This was coupled to a Model 5971A Mass Selective Detector (MSD) (Hewlett-Packard, Palo Alto, CA, USA) operated in the scan mode over the specified mass ranges as specified by EPA Methods 624 or 625, depending on the analysis. A Hewlett-Packard (HP) S/SL injection port was modified as shown in figure 2. First, the carrier gas supply line (normally labelled C) was cut approximately 1 in from the inlet weldment. Using a 1/16 in Swagelok® stainless steel (SS) low volume union, the heated transfer line from a Model 4560 Sample Concentrator (OI Analytical, College Station, TX, USA) was connected, providing the Sample Concentrator interface and helium carrier gas input. For this analysis, an External Carrier Module (ECM) (OI Analytical) supplied a constant gas flow to the GC system. In all states, except the sample concentrator's 'DESORB' state, the carrier gas supply flowed from the mass flow controller through the six-port switching valve in the Model 4560 into the inlet. At this juncture, the gas flow was controlled by adjusting the column head pressure control to allow 1.0 ml/min (at 30°C column temperature) of the total flow into the column. The remainder of the flow was split out of the injector (the injection purge valve was ON) and the septum purge vent was then turned OFF.

The advantage of this flow control scheme is that the total gas flow, supplied by the ECM, desorbs the trap when placed in line with the injector (see figure 3). The higher gas flow needed to efficiently desorb analytes from the trap is provided and the requirement for low gas flow to the MSD is also met. By adjusting the total gas flow, the range, or sensitivity, of the analysis can easily be adjusted. Table 1 shows flow settings used by other analysts when performing similar analyses with the HP Model 5971A MSD (Feyerherm [1]). While performing semi-volatile analyses, a Model 7673 Automatic Injector (HP) was installed over the injector to allow syringe injections. Again, the concentration range of the analysis depended on the split flow.

The introduction of a new volatile organic analysis (VOA) vial sampler, the Model 4551 Vial Multisampler (see figure 4) (OI Analytical), provides the versatility of this system. Table 2 lists several Model 4551 design features. With the introduction of this multisampler and the connection to the 16-station Discrete Purging Multisampler (DPM-16), this GC/MS system is capable of performing VOA on liquids, solids, and air. When combined with the syringe auto-injector, the system is completely automated for both volatile and semi-volatile analyses.

0142-0453/93 $10.00 © 1993 Taylor & Francis Ltd.

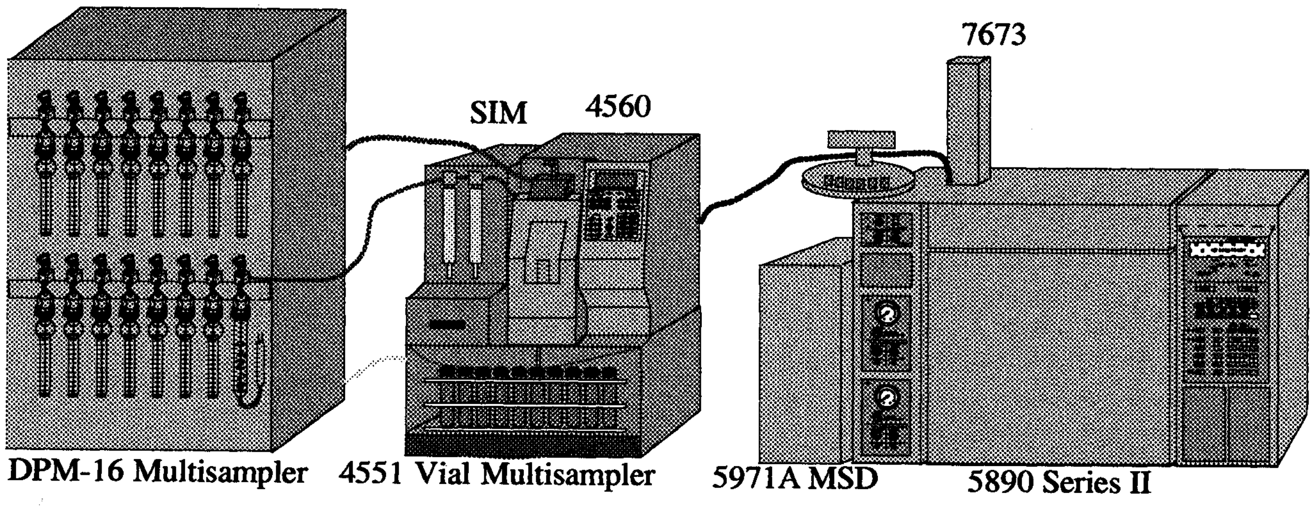

*Figure 1. Multi-tasking GC/MS system.*

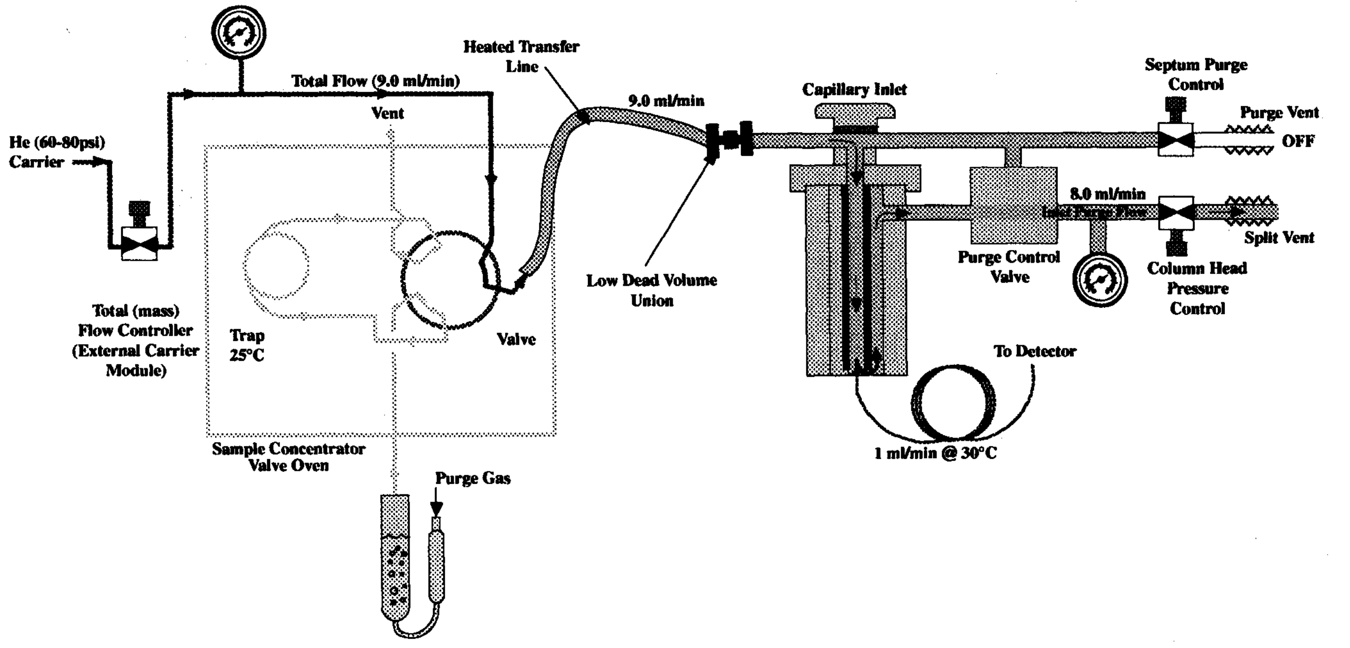

*Figure 2. Sample Concentrator interface to S/SL injector, Sample Concentrator 'PURGE' state.*

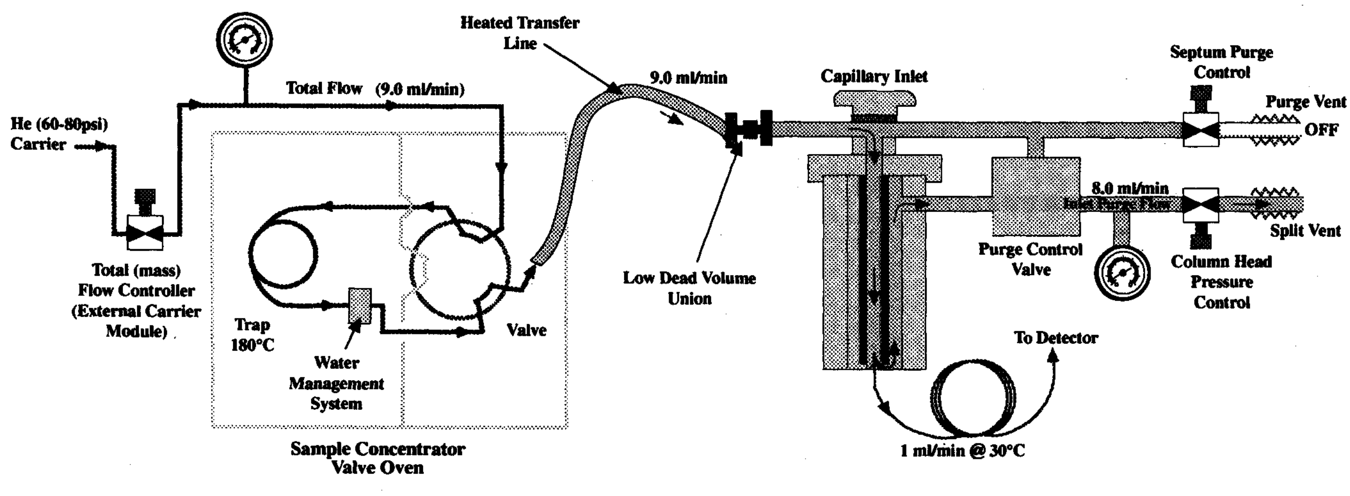

*Figure 3. Sample Concentrator 'DESORB' state.*

*Table 1. Suggested split vent flows for various volatile organic analyses.*

| Calibration range | Split vent flow | Applicable methods |
|---|---|---|
| 20–200 ppb | 50–100 ml/min | 624, 8260, CLP VOAs |
| 0.2–20 ppb | 1–10 ml/min | 524.2, CLP VOAs |
| 0.5–20 ppb | 9.0 ml/min | This analysis |

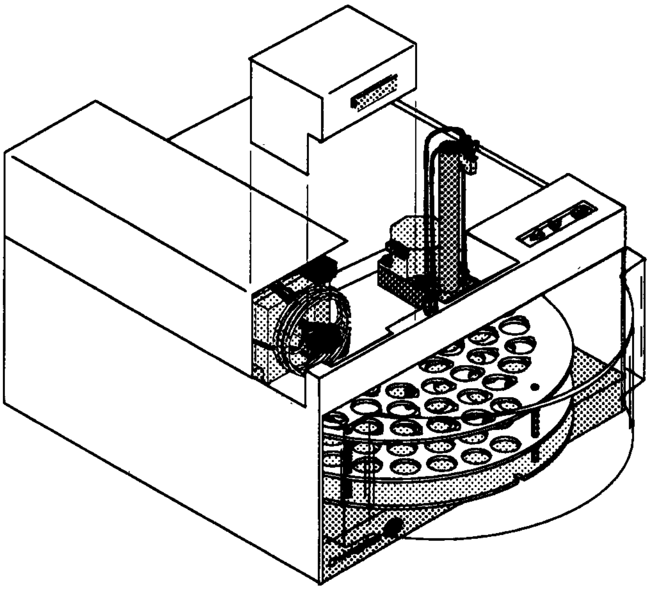

*Figure 4. Model 4551 vial multisampler.*

*Table 2. Design features of the Model 4551.*

- 51 sample positions plus one priority position
- Automatic sample sparger and sample loop rinse
- Internal reagent blank water generation
- Automatic internal standard addition (SIM)
- Automatic surrogate standard addition (Spiker)
- Vial sampling under positive pressure
- Sample syringe depth adjustment
- Interfacing with OI Analytical Discrete Purging Multisampler (DPM-16)

### Experimental

The principle of operation for the Model 4551 is illustrated in figure 5. For this analysis, a 5 ml nickel sample loop was used. The sample is transferred under pressure from the sample vial to overfill the loop volume; the sample is then transferred from the loop through a Standard Injection Module (SIM). As the sample begins to pass through the SIM valve (see figure 6), the module rotates the valve to place a 10 ml gas-tight syringe filled with internal standard solution in line with the valve rotor's internal volume. The SIM advances the syringe plunger, filling the volume of the valve (nominal 10 µl); the rotor rotates back in line with the sample which then sweeps the internal standard solution to the sparge vessel. A second module can inject a surrogate standard solution (Spiker) based on the same valve logic.

*Table 3. Preparation of internal and surrogate standard solutions.*

| Internal surrogate standards | Surrogate standards | Internal standards |
|---|---|---|
| Fluorobenzene | Pentafluorobenzene | Fluorobenzene |
| *p*-Bromofluorobenzene | Toluene-d8 | |
| 1,2-Dichlorobenzene-4 | 1,4-Difluorobenzene | |
| | *p*-Bromofluorobenzene | |
| | 1,4-Dichlorobenzene-d4 | |
| | 1,2-Dichloroethane-d4 | |
| | Chlorobenzene-d5 | |
| | 1-Chloro-3-fluorobenzene* | |

*Prepared at 0.4 ppm for a 5 ml concentration of 0.8 ppb.

Three solutions were used to test these modules with the connecting multisamplers (see table 3). Each of these standards was prepared by adding the appropriate volume of a concentrate stock solution, made up in methanol, to a volumetric flask (typically 250 ml) containing volatile-free water. The concentration of this SIM solution was 2.5 µg/ml of each analyte, which yielded a final concentration of 5 µg/ml when 10 µl of the solution was added to the 5 ml sample. By making the standard solution in water, rather than methanol, the amount of methanol added to the sample by the 10 µl SIM injection resulted in a lower methanol concentration (about 1.5 µg/ml). This is at least 50 times less than the amount of solvent added by EPA procedures when internal or surrogate standards are added and has the effect of reducing the methanol interferences that occur during chromatographic and MSD analysis. The extra standard solution was stored in 40 ml VOA vials with Teflon-faced solid caps at 4°C. The solutions proved to be stable over the two-week test period. A calibration curve was established, based on the internal standard of fluorobenzene from the SIM. The analytes tested are those specified in EPA Method 524.2 (Rev. 3), listed in table 4. The linear response for this list is exemplified by the response for chloroform (see figure 7) with correlation coefficients of greater than 0.997 for all analytes and response ratios within the allowable 20% relative standard deviation (RSD). The column used was a 30 m × 0.25 mm I.D. × 0.25 µdf, DB-5 MS (J&W Scientific, Folsom, CA, USA). This column was more than adequate for the separation of these analytes, even showing separation of all the xylene isomers. However, to obtain good dichlorodifluoromethane (Freon 12) resolution, a subambient oven temperature was used (see figure 8). An initial temperature of −80°C was used in this analysis, although higher temperatures are used when Freon 12 is not a compound of interest. Table 5 shows that excellent retention time (Rt) stability was achieved over the entire range of analytes.

### Results and discussion

Figure 9 shows the response of several phenols analysed according to EPA Method 625. The concentration of each component is 100 µg/l, introduced as a 1 µl injection using the HP Model 7673 Auto Injector. Computer-based, analysis-method sequencing provided the ability to

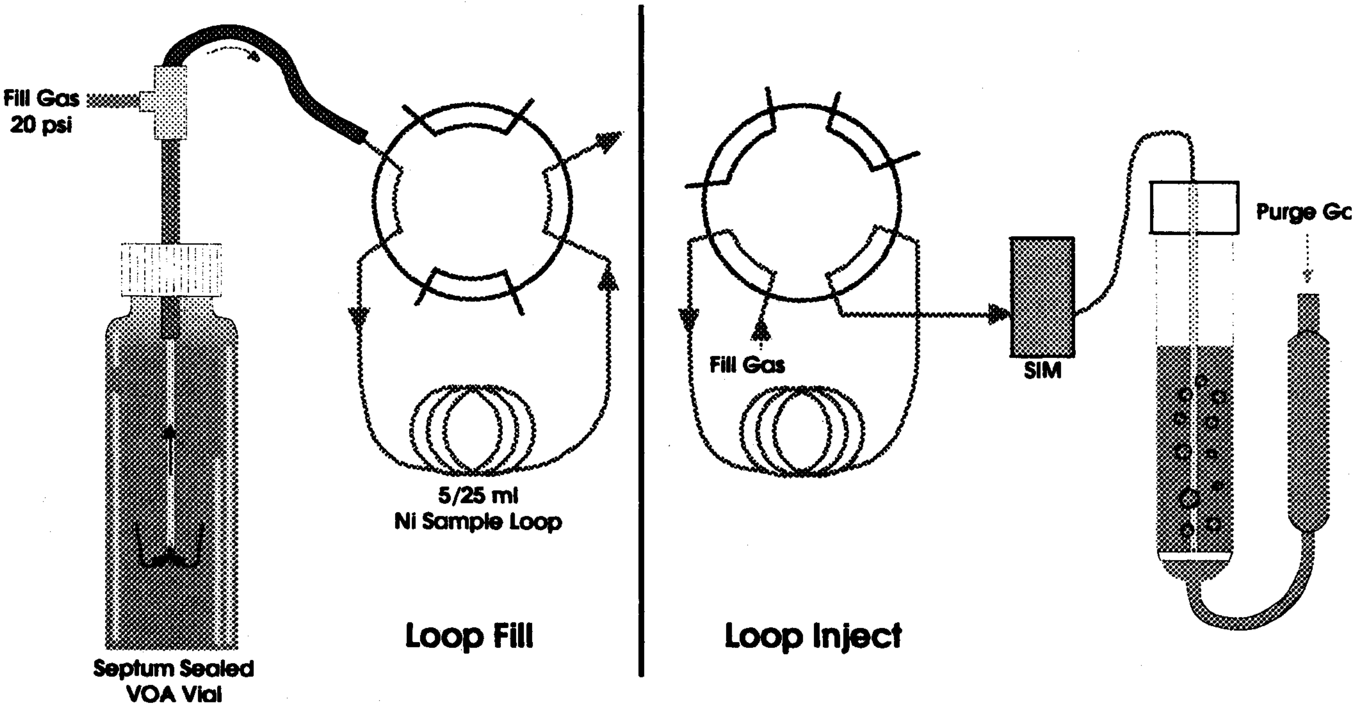

*Figure 5. Model 4551 loop injection sequence.*

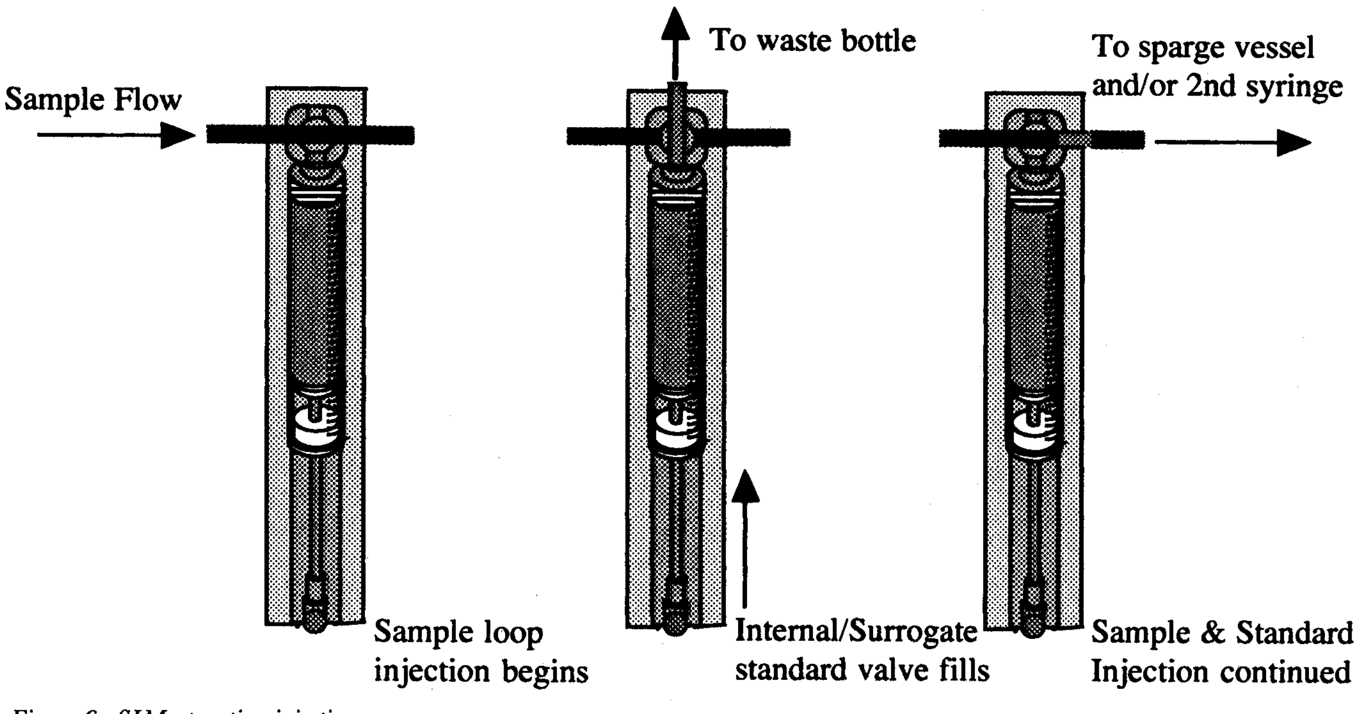

*Figure 6. SIM operation injection sequence.*

configure the GC/MS system for the analysis of syringe injections and purge-and-trap analysis.

Considering all the steps involved in manually preparing a water sample for purge-and-trap analysis (see table 6), the possibility of error or mistakes certainly exists. The fact that the sample vial must be opened to the atmosphere can introduce high and/or low bias results because analytes can be introduced and/or outgased from the sample. By automating these steps, reproducibility and result accuracy are improved.

Table 7 shows the SIM reproducibility during the three-week test period of this analysis with a mixture of the internal standard and surrogates. This standard was added to every sample analysed at an injection interval of 1. Reproducibility is very good for the internal standard and the *p*-Bromofluorobenzene surrogate. The percentage RSD for the 1,2-Dichlorobenzene-$d_4$ was 16%. This high value was first perceived as a hardware problem until further investigation showed good reproducibility for the other surrogates, particularly for 1,4-Dichlorobenzene-$d_4$.

*Table 4. USEPA Method 524.2 (Rev. 3) analytes.*

| | | | | | |
|---|---|---|---|---|---|
| 1 | Dichlorofluoromethane | 22 | 1,2-Dichloropropane | 43 | *p*-Bromofluorobenzene (Surrogate |
| 2 | Chloromethane | 23 | Dibromomethane | | Standard) 5.0 µg/l |
| 3 | Vinyl chloride | 24 | Bromodichloromethane | 44 | Bromobenzene |
| 4 | Bromomethane | 25 | *trans*-1,3-Dichloropropene | 45 | *n*-Propylbenzene |
| 5 | Chloroethane | 26 | *cis*-1,3-Dichloropropene | 46 | 2-Chorotoluene |
| 6 | Trichlorofluoromethane | 27 | Toluene | 47 | 4-Chlorotoluene |
| 7 | 1,1-Dichloroethene | 28 | 1,1,2-Trichloroethane | 48 | 1,3,5-Trimethylbenzene |
| 8 | Methylene chloride | 29 | 1,3-Dichloropropane | 49 | *t*-Butylbenzene |
| 9 | *trans*-1,2-Dichloroethene | 30 | Dibromochloromethane | 50 | 1,2,4-Trimethylbenzene |
| 10 | 1,1-Dichloroethane | 31 | Tetrachloroethene | 51 | sec-Butylbenzene |
| 11 | *cis*-1,2-Dichloroethene | 32 | 1,2-Dibromoethane | 52 | 1,3-Dichlorobenzene |
| 12 | 2,2-Dichloropropane | 33 | Chlorobenzene | 53 | 1,4-Dichlorobenzene |
| 13 | Bromochloromethane | 34 | 1,1,1,2-Tetrachloroethane | 54 | Isopropyltoluene |
| 14 | Chloroform | 35 | Ethylbenzene | 55 | 1,2-Dichlorobenzene-$d_4$ (Surrogate |
| 15 | 1,1,1-Trichloroethane | 36 | *m*-Xylene | | Standard) 5.0 µg/l |
| 16 | 1,2-Dichloropropane | 37 | *p*-Xylene | 56 | 1,2-Dichlorobenzene |
| 17 | 1,1-Dichloropronene | 38 | *o*-Xylene | 57 | *n*-Butylbenzene |
| 18 | Benzene | 39 | Bromoform | 58 | 1,2-Dibromo-3-chloropropane |
| 19 | Carbon tetrachloride | 40 | 1,1,2,2-Tetrachloroethane | 59 | 1,2,4-Trichlorobenzene |
| 20 | Fluorobenzene (Internal Standard) | 41 | Isopropylbenzene | 60 | Naphthalene |
| | 5.0 µg/l | 42 | 1,2,3-Trichloropropane | 61 | Hexachlorobutadiene |
| 21 | Trichloroethene | | | 62 | 1,2,3-Trichlorobenzene |

*Table 5. Purge and trap/split injector rejection times (Rt) reproducibility for selected analytes.*

| Compound | Expected Rt from initial calibration (min) | Two-week average Rt ± SD (min) |
|---|---|---|
| Dichlorodifluoromethane | 3.19 | 3.17 ± 0.01 |
| Vinyl chloride | 3.99 | 3.97 ± 0.005 |
| Chloroform | 7.60 | 7.60 ± 0.005 |
| Fluorobenzene (Internal Standard) | 8.69 | 8.69 ± 0.01 |
| Bromodichloromethane | 9.53 | 9.53 ± 0.00 |
| Toluene | 10.77 | 10.77 ± 0.005 |
| *m*-Xylene | 13.06 | 13.06 ± 0.01 |
| *9*-Xylene | 13.57 | 13.57 ± 0.01 |
| *p*-Bromofluorobenzene | 14.31 | 14.31 ± 0.005 |
| 1,3-Dichlorobenzene | 15.99 | 15.99 ± 0.01 |
| Naphthalene (Split ratio ≈ 10:1) | 19.51 | 19.51 ± 0.01 |

*Table 6. Summary of sample injection steps for VOC analysis.*

1 Open sample vial
2 Pour sample into gas-tight syringe barrel
3 Inject internal standard into gas-tight syringe
4 Inject surrogate standard into gas-tight syringe
5 Inject sample into sparge vessel
6 Before next sample, rinse all glassware (twice)

*Table 7. Reproducibility of internal standard and surrogate standard using SIM.*

| | |
|---|---|
| Fluorobenzene | 6.6% RSD |
| *p*-Bromofluorobenzene | 4.7% RSD |
| 1,2-Dichlorobenzene-$d_4$ | 16% RSD |

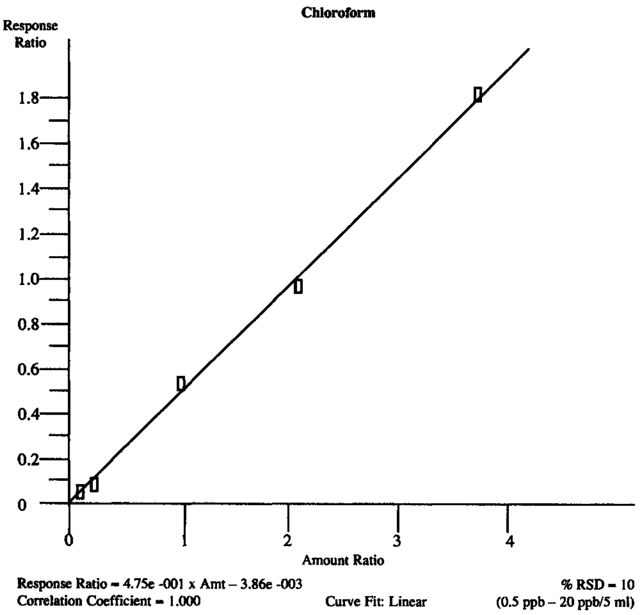

*Figure 7. Response to chloroform.*

Also, the relatively poor percentage RSD value was a result of low responses and varied with the presence or absence of the hydrogenated isomer, 1,2-Dichlorobenzene. A possible explanation is de-deuteration due to hydrogen exchange either in the MSD ion source, or during the gas phase of the analysis. This occurrence will be investigated further in future analyses.

Table 8 shows the reproducibility of a SIM and a Spiker, a second SIM module that has been set to inject a standard solution at an alternating interval between 0–99 (interval = 2 for this analysis). The Spiker option can be used to add surrogates to a sample or blank, or to hold a second volume of the internal standard solution. The Spiker option can then be sequenced by the Model 4560 microprocessor to

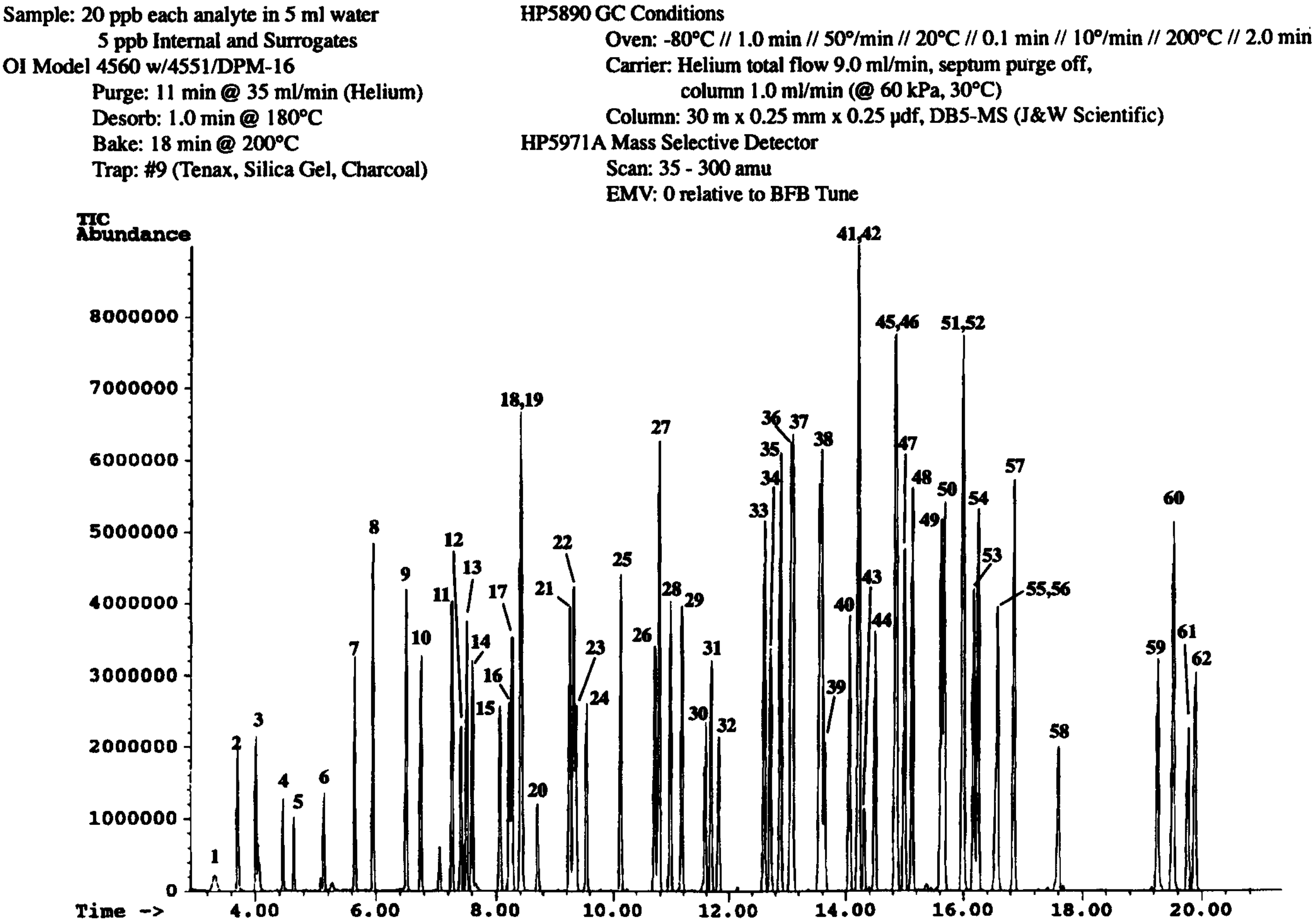

*Figure 8. GC/MS analysis of EPA Method 524.2 analytes with split injector interface.*

*Table 8. Reproducibility of surrogate injections.*

| Compound (5 μg/l) | Amount detected (μg/l)* | % RSD |
|---|---|---|
| Pentafluorobenzene | 4.7 | 6.0 |
| 1,2-Dichloroethane-$d_4$ | 5.0 | 3.0 |
| 1,4-Difluorobenzene | 4.9 | 4.1 |
| Toluene-$d_8$ | 5.0 | 3.3 |
| 1-Chloro-3-fluorobenzene** | 0.3 | 5.8 |
| Chlorobenzene-$d_5$ | 4.9 | 3.5 |
| *p*-Bromofluorobenzene | 5.0 | 3.4 |
| 1,4-Dichlorobenzene-$d_4$ | 4.8 | 5.0 |

*Mean, $n = 19$.

**0.4 μg/l.

*Table 9. Reproducibility of 4551 replicates.*

| Level (μg/l) | Average % RSD | Range % RSD |
|---|---|---|
| 0.5 | 3.8 | 0–15 |
| 1.0 | 1.1 | 0–10 |
| 5.0 | 3.5 | 0–5.0 |
| 10 | 2.6 | 0–5.2 |
| 20 | 1.4 | 0–5.1 |

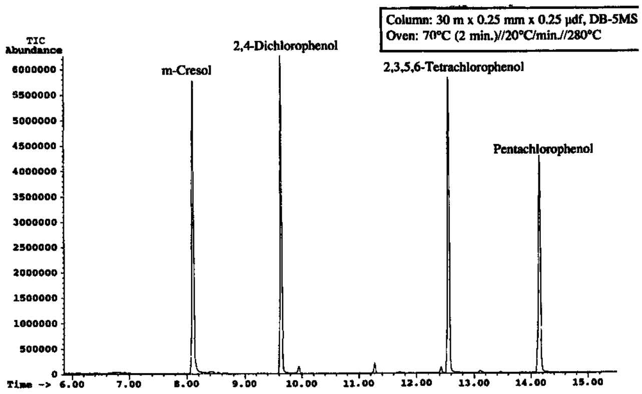

*Figure 9. Analysis of selected phenols.*

turn ON when the primary syringe empties (about 100 injections).

With a 5 ml sample loop, it is possible to analyse multiple replicates for a 40 ml VOA vial. By adjusting the loop fill time to a minimum and adjusting the needle depth, all but the final 1 ml of water can be sampled. Table 9 shows the reproducibility of two replicates taken across the calibration range.

The usefulness of any quantitative analysis automatic sampling system is limited by the amount of carryover

*Table 10. Carryover for purge-and-trap multisamplers.*

| Sampling technique | Average % carry-over | Range (μg/l) | Comments |
|---|---|---|---|
| 4551 vial sampler 20 μg/l (two rinses) | 0.51 | 0–4.0 | Tetrachloroethene only compound detected |
| 4551 vial sampler 200 μg/l (three rinses) | 0.01 | 0–0.1 | Benzene, Hexachlorobutadiene, Naphthalene, only compounds detected |
| 4551 vial sampler to DPM-16 200 μg/l (two rinses) | 0.26 | 0–1.3 | Naphthalene @ 1.3 μg/l all others < 0.6 μg/l |
| DPM-16 200 μg/l | 0.35 | 0–1.4 | Methylene chloride @ 1.4 μg/l Naphthalene @ 1.2 μg/l Tetrachloroethene @ 0.9 μg/l all others < 0.6 μg/l |

experienced when a high level sample is encountered. Table 10 shows the results of four tests performed on the purge-and-trap multisamplers. The first two tests were for the Model 4551 connected directly to the Model 4560 Sample Concentrator. Results are for a blank analysed after a 20 μg/l (top row) and a 200 μg/l (second row) 524.2 standard. The third row shows carryover following a 200 μg/l sample when the DPM-16 station 16 was used as the sparging location for the Model 4560. The final result is for the DPM-16 station 11 (200 μg/l sample) to station 12 (blank) carryover. These results indicate the Multisamplers' ability to provide meaningful results in a given calibration range.

### Conclusion

A multi-tasking GC/MS system is useful to both laboratories operating on a limited capital budget, and laboratories needing more flexibility. This analysis has demonstrated the abilities of the Model 4551/DPM-16 Multisampler interface used in conjunction with the Model 4560 Sample Concentrator. It has also been demonstrated that with a relatively simple injector modification and a proper capillary column, a GC/MS system can be configured to analyse both volatile and semi-volatile compounds.

### Reference

1. Feyerherm, F., Hewlett-Packard Co., Houston, Texas, USA, personal communication, 1990.

